# Identification of Cisplatin-Binding Proteins Using Agarose Conjugates of Platinum Compounds

**DOI:** 10.1371/journal.pone.0066220

**Published:** 2013-06-03

**Authors:** Takatoshi Karasawa, Martha Sibrian-Vazquez, Robert M. Strongin, Peter S. Steyger

**Affiliations:** 1 Oregon Hearing Research Center, Oregon Health & Science University, Portland, Oregon, United States of America; 2 Department of Chemistry, Portland State University, Portland, Oregon, United States of America; University of Cambridge, United Kingdom

## Abstract

Cisplatin is widely used as an antineoplastic drug, but its ototoxic and nephrotoxic side-effects, as well as the inherent or acquired resistance of some cancers to cisplatin, remain significant clinical problems. Cisplatin's selectivity in killing rapidly proliferating cancer cells is largely dependent on covalent binding to DNA via cisplatin's chloride sites that had been aquated. We hypothesized that cisplatin's toxicity in slowly proliferating or terminally differentiated cells is primarily due to drug-protein interactions, instead of drug-DNA binding. To identify proteins that bind to cisplatin, we synthesized two different platinum-agarose conjugates, one with two amino groups and another with two chlorides attached to platinum that are available for protein binding, and conducted pull-down assays using cochlear and kidney cells. Mass spectrometric analysis on protein bands after gel electrophoresis and Coomassie blue staining identified several proteins, including myosin IIA, glucose-regulated protein 94 (GRP94), heat shock protein 90 (HSP90), calreticulin, valosin containing protein (VCP), and ribosomal protein L5, as cisplatin-binding proteins. Future studies on the interaction of these proteins with cisplatin will elucidate whether these drug-protein interactions are involved in ototoxicity and nephrotoxicity, or contribute to tumor sensitivity or resistance to cisplatin treatment.

## Introduction

Cisplatin (*cis*-diamminedichloroplatinum[II]) is widely used in chemotherapy to treat various cancers with high efficacy, with remission rates of over 90% in testicular cancers [1]. More than one million patients receive platinum agents in North America and Europe each year. However, cisplatin therapy is limited by intrinsic and acquired tumor resistance, and severe side-effects in specific tissues [2]. Over 60% of cisplatin-treated pediatric patients develop irreversible hearing loss [3], [4], and more than 70% experience acute renal dysfunction [5]. Pediatric hearing loss delays language acquisition, educational attainment and social integration [3], [6].

**Figure 1:**
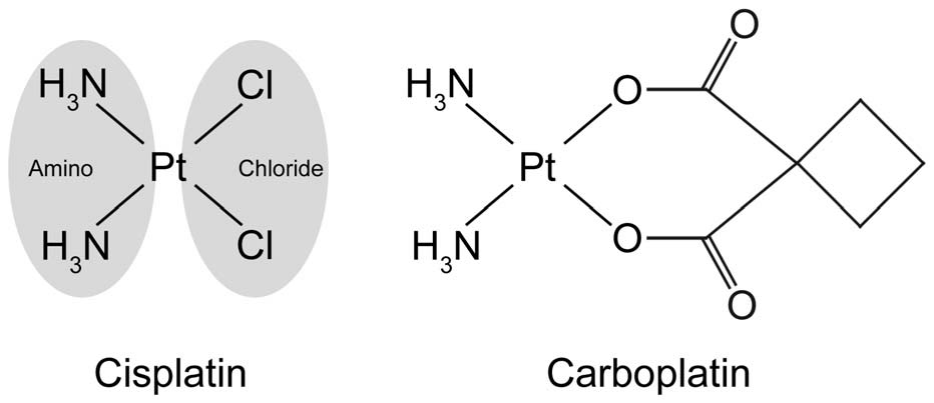
Structures of cisplatin and carboplatin.

Cisplatin induces DNA damage and cell death in rapidly-proliferating cells, especially cancer cells. This involves aquation of the two chlorides (Fig. 1), and subsequent binding to DNA, forming DNA adducts [7]–[9]. Cells recognize this as DNA damage, and induce various signaling cascades, leading to cell cycle arrest, apoptosis, or DNA repair [10]. However, kidney proximal tubule cells and cochlear cells have low rates of cell proliferation (or none in the case of post-natal cochlear hair cells), yet these cells are especially prone to cisplatin-induced cytotoxicity. Oxidative stress induced by cisplatin has been suggested as a major factor in ototoxicity and nephrotoxicity [11]–[13]. This also involves the conversion of cisplatin into highly-reactive aquated forms that rapidly bind to thiol-containing antioxidants, including glutathione [14]. Cisplatin interaction with antioxidants leads to the accumulation of endogenous reactive oxygen species, which in turn act on many targets in the cell, including lipids, proteins, and DNA, inducing cytotoxicity. Several anti-oxidants including D-methionine, *N*-acetylcysteine, and sodium thiosulphate can prevent platinum-induced ototoxicity [15]–[17].

Carboplatin (Fig. 1), a less toxic analog of cisplatin that is used for some types of cancer, does not readily form platinum-DNA adducts in the cochlea and kidney [18], because its six-membered ring confers greater chemical stability, reducing its conversion rate to aquated platinum species [19]. Although carboplatin is less ototoxic and nephrotoxic compared to cisplatin, carboplatin-induced cytotoxicity still occurs, and unwanted side-effects remain problematic at the doses required for clinical efficacy [20], [21]. It is possible that these side-effects are caused by non-oxidative stress mechanisms because of the greater chemical stability in carboplatin compared to cisplatin.

Since both cisplatin and carboplatin have two amino groups attached to platinum (Fig. 1), these drugs could induce cytotoxicity through molecular interactions at the amino groups. We hypothesized that there are proteins that specifically bind to these two amino groups. This drug-protein interaction could induce toxicity, and trigger cell death mechanisms. In this study, we synthesized two different platinum complexes, one with two amino groups and the other with two chlorides covalently coordinated to platinum and available for protein binding. We conducted protein pull-down assays using agarose conjugates of these complexes, and identified the proteins that bind to cisplatin at the amino groups by mass spectrometric analysis.

## Materials and Methods

### Synthesis of Pt complexes and their agarose conjugates

#### General

Unless otherwise indicated, all commercially available starting materials were used directly without further purification. Silica gel, Sorbent Technologies 32–63 µm was used for flash column chromatography. ^1^H-NMR, ^13^C-NMR and ^195^Pt-NMR were obtained on an ARX-400 Avance Bruker spectrometer. ^15^N-NMR was obtained on an ARX-600 Avance Bruker spectrometer. Chemical shifts (δ) are given in ppm relative to TMS or DSS unless otherwise indicated. For ^195^Pt-NMR, K_2_PtCl_4_ was used as external standard. For ^15^N-NMR, ^15^NH_4_Cl was used as external standard. MS (HRMS, ESI) spectra for all synthesized compounds were obtained at the PSU Bioanalytical Mass Spectrometry Facility on a ThermoElectron LTQ-Orbitrap high resolution mass spectrometer with a dedicated Accela HPLC system.

**Synthesis of N^3^-(tert-Butoxycarbonyl)-1,2,3-propanetriamine, 1.** The title compound was synthesized from 3-amino-1,2-propanediol as described previously [22], in three steps and in an overall yield of 73%. NMR and MS structural characterization are in agreement with the published data.

**Synthesis of N-(tert-Butoxycarbonyl)-1-aminomethyl-1,2 ethylenediamine)dichloroplatinum(II), 2.** Compound **2** was synthesized using a modified procedure reported previously [23]. Briefly, under Ar atmosphere K_2_PtCl_4_ (1.1 g, 2.64 mmol) was dissolved in 50 ml of DI water. The Boc-protected diamine **1** (0.5 g, 2.64 mmol) was dissolved in 10 ml of DI water and added to the K_2_PtCl_4_ solution under Ar. The mixture was stirred at room temperature for 24 h, after which a pale yellow solid formed. The solid was filtered, washed with water (5×50 ml), and dried under vacuum (Yield 0.9 g 75%). NMR and MS structural characterization are in agreement with the published data.

**Synthesis of (1-aminomethyl-1,2-ethylenediamine)dichloroplatinum(II) hydrochloride, 3.** Compound **3**, was synthesized by a modified procedure reported previously [23]. Compound **2** (500 mg, 1.10 mmol) was suspended in 50 ml of 0.1M HCl, followed by the addition of 2.5 ml of 1M HCl. The reaction mixture was heated at 50°C for 24 h, then 0.5 ml of 1M HCl was added and the reaction mixture heated at 50°C for additional 24 h. The mixture was allowed to cool down to room temperature and filtered through a 0.22 µm syringe filter to remove a white precipitate. The filtrate was evaporated under vacuum and the yellow residue dried under vacuum (Yield 410 mg, 96%). NMR and MS structural characterization are in agreement with the published data.

**Synthesis of (1-aminomethyl-1,2-ethylenediamine)diamineplatinum(II), 5.** Compound **3** (200 mg, 0.512 mmol) was suspended in 5 ml of Milli-Q type water, then the mixture was heated and stirred at 40°C for 4 h. NH_4_OH (1.28 mmol) was added in one portion and the reaction mixture heated at 40°C for additional 2 h. The mixture was allowed to cool down to room temperature, then additional NH_4_OH (1.28 mmol) was added and the mixture allowed to stand for 3 h without stirring. The solvent was evaporated under vacuum to leave a yellow residue (Yield 159.5 mg, 98%). ^1^H NMR (400 MHz, D_2_O) δ 2.64–2.74 (1H, m), 3.02–3.12 (2H, m), 3.28–3.41 (2H, m). ^13^C NMR (101 MHz, D_2_O) δ 48.76, 46.01, 38.68. ^195^Pt NMR (101 MHz, D_2_O) δ −2757.04, −2782.69. HR ESI MS, *m/z* [M]^+^ 317.1058; calc. for C_3_H_17_N_5_Pt, 317.1105; *m/z* [M]^2+^ 158.5556; calc for C_3_H_17_N_5_Pt; 158.5550, *m/z* [M-NH_3_]^+^ 300.0790; calc. for C_3_H_17_N_5_Pt, 301.0866; *m/z* [M-NH_3_]^2+^ 150.5435, calc. for C_3_H_17_N_5_Pt 150.5433.

### Synthesis of 2ClPt-agarose and 2NH_3_Pt-agarose conjugates

Each of the Pt complexes **3** and **5** was dissolved at 10 mg/ml in 0.1 M NaHCO_3_ (pH 8.3). 1 ml of the Pt complex solution was added to 100 µl of Affi-Gel 10 (Bio-Rad, Hercules, CA). The mixture was incubated at 4°C overnight. The residual active esters on the gel were blocked with 1 M ethanolamine (pH 8.0) for 1 h. The Pt-agarose conjugate was washed with cold 0.1 M NaHCO_3_ (pH 8.3) to remove unbound Pt complex.

#### Cell culture

The mouse organ of Corti HEI-OC1 and stria vascularis SV-k1 cells have been previously described [24], [25] (generous gifts from Dr. Federico Kalinec, House Research Institute), and were maintained at 33°C with 10% CO_2_ [26]. Mouse kidney proximal tubule KPT2 and KPT11, and distal tubule KDT3 as previously described [26], [27], hepatocyte AML12 (ATCC), embryonic fibroblast K41 [28], and human embryonic kidney 293T cells (ATCC) were maintained at 37°C with 5% CO_2_. All cells were maintained in growth medium (DMEM with 10% FBS).

#### Pt-agarose pull-down assay

Cells were lysed in buffer containing 150 mM NaCl, 50 mM Tris, pH 7.5, 5 mM EDTA, 1% Triton X-100 with protease inhibitor cocktail (Sigma), and centrifuged (14,000 *g*, 10 min) at 4°C to remove cell debris. Protein concentration was adjusted to 2 mg/ml for all samples. The Pt-agarose conjugate was incubated with whole cell extract (1 ml) for 2 h at 4°C. Beads with bound proteins were spun down by brief centrifugation, and washed with the same lysis buffer at 4°C. The proteins were removed from Pt-agarose in SDS sample buffer, boiled for 2 min, and separated by protein gel electrophoresis (8% gel) prior to Coomassie blue staining. For pull-down of FLAG-tagged HSP90 proteins (FLAG-HSP90), 293T cells transfected with FLAG-tagged HSP90α or HSP90β were lysed in lysis buffer containing glycerol at 10% final concentration for cell lysate preparation, and 50 µl Affi-Gel 10 and 500 µl of Pt complex solution (5 mg Pt dissolved) were used to synthesize 2ClPt-agarose or 2NH_3_Pt-agarose. After ethanolamine treatment, the beads were further blocked with 5% bovine serum albumin (BSA)-containing phosphate-buffered saline (PBS) for 3 h at 4°C, prior to adding cell lysate 1 ml (400 µg/ml) for protein binding.

#### Mass spectrometry

Mass spectrometric analysis was performed by the Proteomics Shared Resource facility at Oregon Health & Science University. Briefly, Pt-binding protein bands were excised from a Coomassie-stained gel, washed twice in 50 mM ammonium bicarbonate/50% (v/v) acetonitrile and dried. The samples were reduced in 10 mM DTT/100 mM ammonium bicarbonate, alkylated in 55 mM iodoacetamide/100 mM ammonium bicarbonate, and dehydrated gels re-swelled in digestion buffer containing 50 mM ammonium bicarbonate/5 mM CaCl_2_/12.5 ng/ µl sequencing grade modified trypsin (Promega, Madison, WI). Following an overnight incubation at 37°C, extracted peptides were identified by collection of MS/MS data using a Velos Pro linear ion trap mass spectrometer (ThermoFisher, San Jose, CA). Peptides were separated using an Agilent 1100 series capillary LC system (Agilent Technologies Inc, Santa Clara, CA) and 0.5×250 mm Zorbax SB-C18 column (Agilent Technologies). Proteins were identified by collection of data-dependent MS/MS spectra and using both Mascot (Matrix Science, London, UK) and Sequest (ThermoFisher) was used to compare spectra to a mouse only protein database (SwissProt, Swiss Institute of Bioinformatics, downloaded on March 1st 2012) with trypsin specificity. The database was also amended with an equal number of decoy sequence-reversed entries to independently assess the protein false discovery rate. Protein identification was then validated using the program Scaffold (Proteome Software, Inc., Portland, OR) which uses a probabilistic model to estimate the peptide and protein false discovery rates [29], [30], and the proteomic analysis workflow (PAW) pipeline to maximize peptide identification while controlling both peptide and protein false discovery rates [31]. Minimum peptide and protein probabilities were set at 80% and 99%, respectively, with a minimum of 5 unique peptide matches per protein. Using these criteria, there were no matches to the decoy proteins with reversed sequences.

#### Plasmid constructs, transfection and retroviral trunsduction

For FLAG-HSP90 expression constructs, rat HSP90α and HSP90β cDNA from Open Biosystems (Clone ID 7114546 amd 7131767, respectively) were amplified by PCR with primers 5′-AAAGCGGCCGCGCCTGAGGAAACCCAGACC-3′ and 5′-CCCGTCGACTAGTCTACTTCTTCCATG-3′ for HSP90α, and 5′-AAAGCGGCCGCGCCTGAGGAAGTGCACCATG-3′ and 5′-CCCGTCGACTAATCCACTTCTTCCATG-3′ for HSP90β, digested with NotI/SalI, and subcloned into pFLAG-CMV2 vector. The resultant plasmids were transfected into 293T cells using Lipofectamine 2000 (Invitrogen). For the plasmid to generate stable cell lines that express HSP90α, primers 5′-AAATACGTACCGCCATGCCTGAGGAAACCCAGACC-3′ and 5′-CCCGTCGACTAGTCTACTTCTTCCATG-3′ were used to amplify HSP90α by PCR, and the PCR product was digested by SnaBI/SalI and subcloned into pBabe-puro vector. The resultant plasmid was transfected into Phoenix Eco packaging cell using Lipofectamine 2000. After 48 h, the retrovirus-containing medium was collected, diluted (1∶500) with growth medium and added to KPT2 cells in culture. Growth medium was changed again after 24 h and puromycin was added at 2.5 µg/ml to select for retrovirus-infected cells. From dozens of surviving cells after several days of puromycin treatment, several clones were selected and expanded.

#### Western blotting

Proteins in lysis buffer were mixed with SDS sample buffer, resolved in SDS-polyacrylamide gel, and transferred to polyvinylidene fluoride membrane. After blocking with 5% skim milk in Tris-buffered saline (TBS), HSP90α rabbit polyclonal antibody (Lab Vision Cat #RB-119), HSP90β rabbit polyclonal antibody (Lab Vision Cat #RB-118), FLAG mouse monoclonal antibody (M2, Sigma), or actin rabbit polyclonal antibody (Sigma, Cat #A2103) was incubated overnight at 4°C. After horseradish peroxidase conjugated secondary antibody incubation for 1 h, chemiluminescence with SuperSignal West Dura Extended Duration Substrate (Pierce) was used to detect protein expression.

#### MTT assay

Cell growth and viability were determined by the reduction of 3-(4,5-dimethylthiazol-2-yl)-2,5-diphenyltetrazolium bromide (MTT), an indicator of mitochondrial dehydrogenase activity. Cells were plated at 3000 cells per well in a 96-well plate. After incubation overnight to allow cells to attach to the plate, cells were treated with cisplatin in culture medium for 2 days. 20 µl of 5 mg/ml MTT solution was added to each well, and incubated for 4 h at 37°C, 5% CO_2_. Culture medium was then replaced with 200 µl dimethyl sulfoxide (DMSO) in each well and the optical density recorded at 540 nm with background subtraction at 660 nm. Student's *t*-test was used for statistical analysis.

## Results

### Synthesis of Pt complexes bearing a free amino group for conjugation to agarose

**Figure 2:**
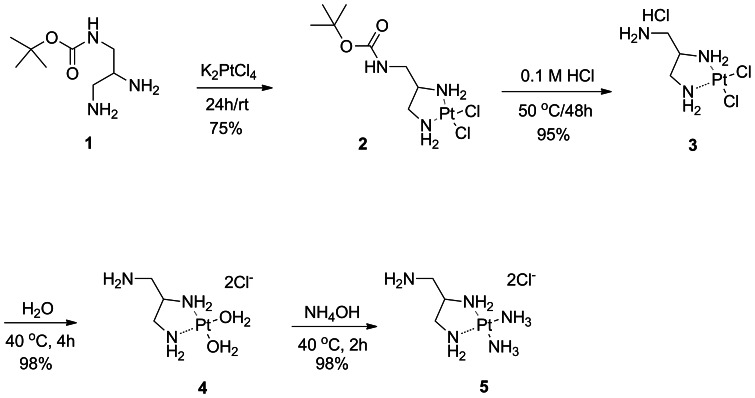
Synthesis of Pt complexes 3 and 5.

**Figure 3:**
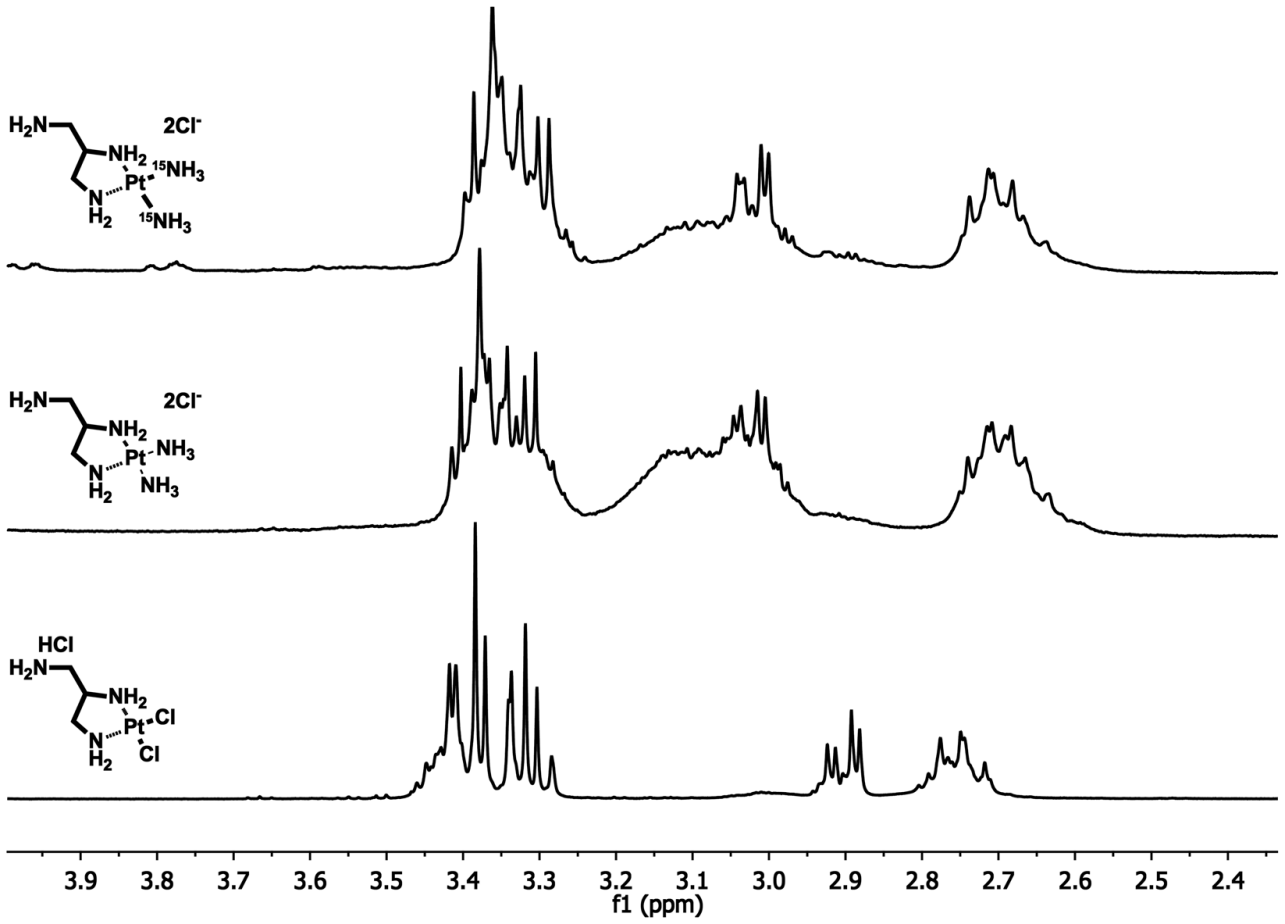
^1^H NMR of Pt complexes. ^1^H NMR spectra in D_2_O shows methylene and methine proton shifts in complex **5** as compared to that of **3**. ^15^N-labeled (top) and non-labeled (middle) complex **5** exhibit similar chemical shifts and splitting patterns.

**Figure 4:**
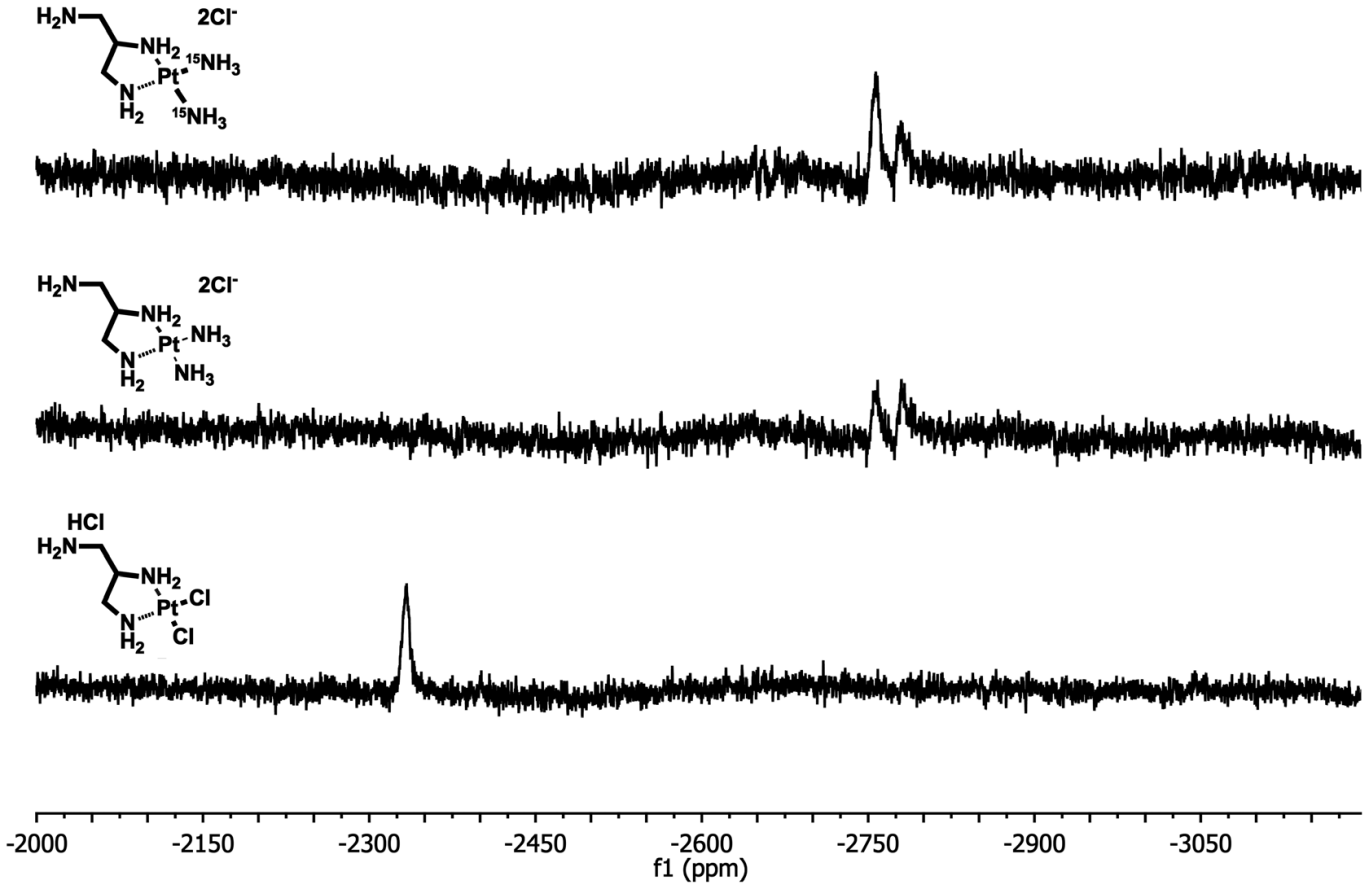
^195^PtNMR spectra of complexes 3 and 5 in D_2_O. Pt complex **5** shows an upfield chemical shift as compared to that of **3**. Similar chemical shifts are observed for the ^15^N-labeled (top) and non-labeled (middle) complex **5**.

**Figure 5:**
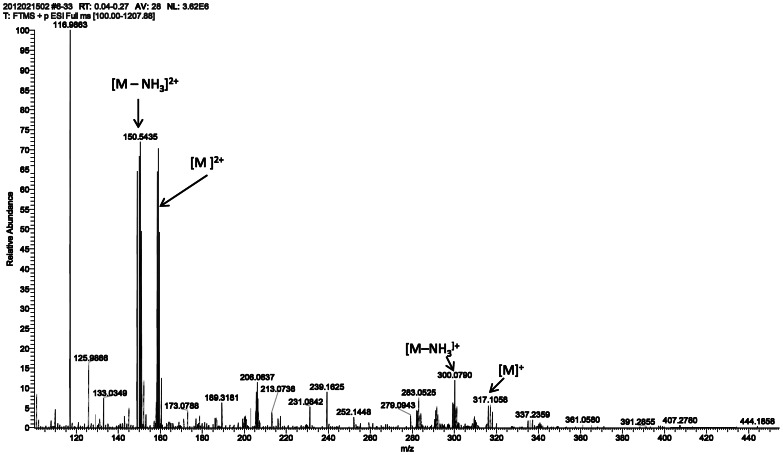
HR ESI positive mode MS spectrum of complex 5.

Two Pt-complex-agarose conjugates were synthesized and designated as 2ClPt-agarose and 2NH_3_Pt-agarose. The corresponding (1-aminomethyl-1,2-ethylenediamine)dichloroplatinum(II) hydrochloride (complex **3**) and (1-aminomethyl-1,2-ethylenediamine)diamineplatinum(II) (complex **5**), bearing an amino group for conjugation to agarose were synthesized as shown in Fig. 2. Complex **3** was synthesized in 52% overall yield by following modified literature procedures [22], [23]. The Pt complex **5** was obtained in 98% yield from **3**, via the formation of the di-aquo complex **4**, followed by ligand exchange with an excess of NH_4_OH [32], [33]. Structural characterization of Pt complexes **3** and **5** was performed by NMR and MS. ^1^H NMR (Fig. 3) shows that there is a significant shift for the methylene and methine protons of **5** as compared to those of **3**; in addition the ^195^Pt NMR spectra (Fig. 4) shows that the Pt complex **5** exhibits the expected upfield chemical shift (−2757.04, −2782.69 ppm) as compared to that of **3**, (−2333.72 ppm) as a result of the Cl→NH_3_ ligand exchange. The upfield chemical shift observed for compound **5** is also in agreement with the δ (^195^Pt) value reported for the analog *cis*-[Pt(ethylendiamine)(NH_3_)_2_]Cl_2_ at −2795 ppm [34]. In order to further confirm the Cl→NH_3_ ligand exchange, a ^15^N-labeled Pt complex was also synthesized using labeled ^15^NH_4_OH. ^1^H NMR and ^195^Pt NMR spectra of both non-labeled and ^15^N-labeled Pt complexes **5** exhibited similar chemical shifts and splitting patterns (Figs. 3 and 4). In addition, ^15^N-HIG and ^15^N-H HSQC NMR experiments suggest that at least one ^15^NH_3_ is coordinated to the Pt atom since a peak with a complex splitting pattern is observed at −47.2 ppm (data not shown). The HR ESI positive mode mass spectrum (Fig. 5) of compound **5** shows the characteristic isotopic pattern of Pt for the parent ion *m/z* calculated for [M]^+^ C_3_H_17_N_5_Pt; 317.1105; *m/z* observed; 317.1058 and for its corresponding [M]^2+^ ion; *m/z* calculated for C_3_H_17_N_5_Pt; 158.5550; *m/z* observed; 158.5556. A second signal of same intensity as for the [M]^2+^ ion could be assigned as the [M-NH_3_]^2+^ fragment; *m/z* calculated for C_3_H_14_N_4_Pt; 150.5433 *m/z* observed; 150.5435, this could be associated to the peak observed with a *m/z* of 300.0790; calculated for [M-NH_3_]^+^ 301.0866.

### 2NH_3_Pt-agarose binds to proteins with higher affinity compared to 2ClPt-agarose

**Figure 6:**
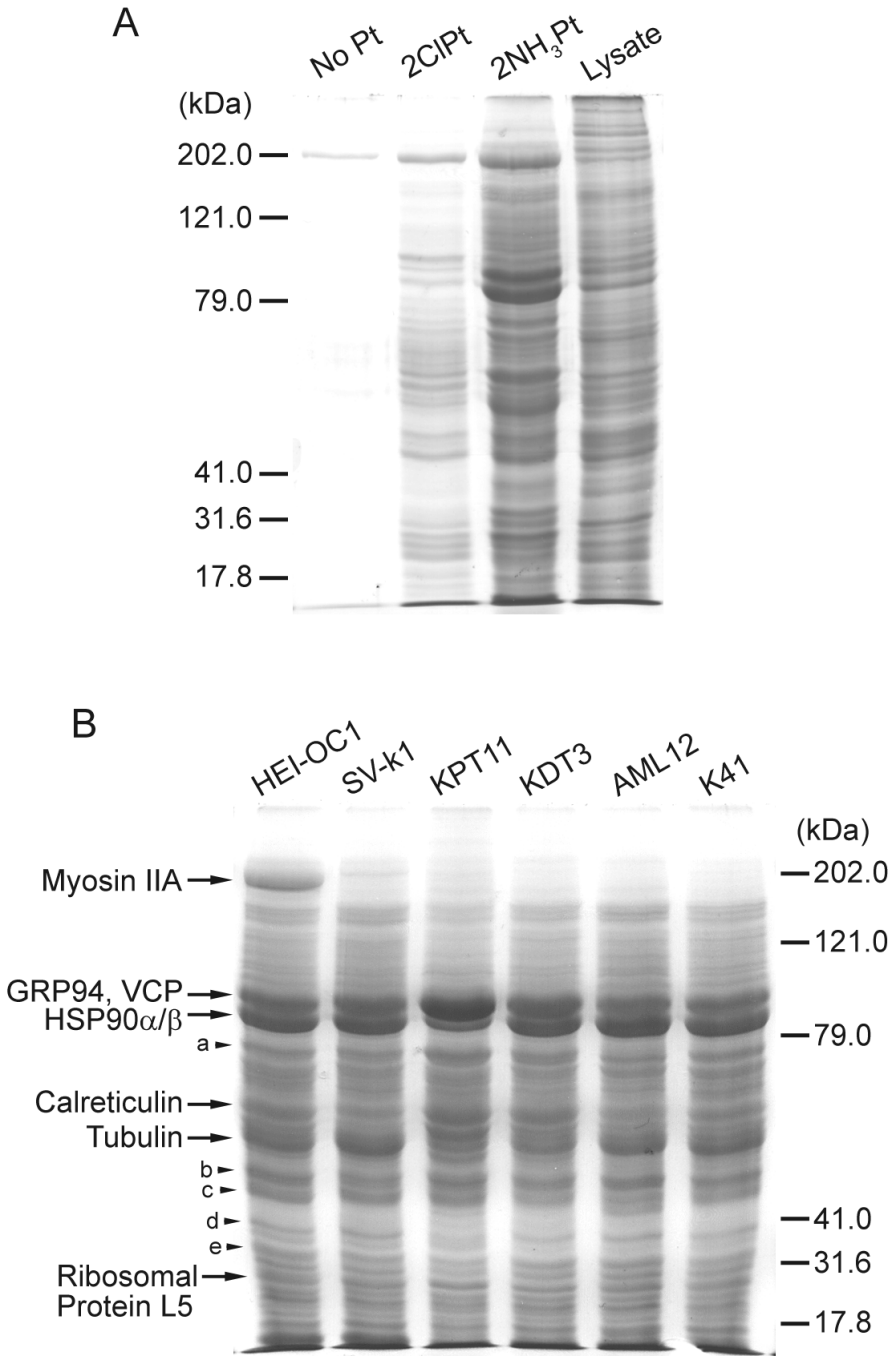
Cisplatin-binding protein pull-down assay results. (A) Pull-down assay samples using HEI-OC1 cells. Agarose without Pt conjugation (No Pt) was used as a control. 2NH_3_Pt-agarose pulled down several proteins in significant amounts, while 2ClPt-agarose only pulled down fewer proteins in lower amounts. The total cell lysate (Lysate) sample before pull-down is to show relative expression levels of proteins with different molecular mass. (B) 2NH_3_Pt-agarose pull-down assay results. Several protein bands with high intensity appeared in all cells tested, with some minor band differences in different lanes. Gel bands were excised from HEI-OC1 samples and analyzed by mass spectrometry. Protein names identified in gel regions a, b, c, d and e are shown in Table 1.

We conjugated Pt complexes **3** and **5** to agarose to produce the corresponding 2ClPt-agarose and 2NH_3_Pt-agarose conjugates. These conjugates were used to perform protein pull-down assays using total cell lysate from mouse organ of Corti HEI-OC1 cells, similar to that described previous for gentamicin-binding proteins [26], [28]. The bound protein samples were removed from Pt-agarose and analyzed by SDS-gel electrophoresis and Coomassie blue staining. Many protein bands, including several intense bands, were detected in the sample from 2NH_3_Pt-agarose, while fewer, less intense bands were detected in the sample from 2ClPt-agarose (Fig. 6A).

### Identification of cisplatin-binding proteins

Using the 2NH_3_Pt-agarose, we pulled down proteins from cell lysate of several different mouse cell lines. Because of our interest in ototoxicity and nephrotoxicity induced by cisplatin, we used two cochlear cell lines (HEI-OC1 and SV-k1), and two kidney cell lines (KPT11 and KDT3). We also used murine AML12 cells (liver hepatocytes) and K41 cells (mouse embryonic fibroblasts). As shown in Fig. 6B, all binding protein samples exhibited similar band patterns, although some differences in band intensity patterns could be detected among different cell lines, including a strong band around 200 kDa (myosin IIA) in only HEI-OC1 cells and a band around 85 kDa (HSP90α/β) that is much weaker in KPT11 (Fig. 6B). The band around 55 kDa (tubulin) was also low intensity in kidney cells compared to the other cells. We excised protein bands with high intensities from the HEI-OC1 sample and analyzed each band by mass spectrometry, because these bands are likely specific binding proteins to cisplatin's amino groups, and these same bands were weak or non-existent in pull-down samples from 2ClPt-agarose or agarose alone. The most abundant proteins identified were non-muscle myosin IIA, glucose-regulated protein of 94 kDa (GRP94), valosin-containing protein (VCP), heat shock protein 90α and β (HSP90α/β), calreticulin, tubulin (seven isoforms: α1a, β2a, β2b, β3, β4b, β5, and β6), and 60S ribosomal protein L5. Additional gel band analysis by mass spectrometry identified several more proteins (a, b, c, d, and e in Fig. 6B), and the mass spectrometry results are shown in Table 1. The histidine content in each identified protein is also included in Table 1, since recent reports suggest that histidine residues in a protein contribute to cisplatin binding [35], [36]. While most of the proteins have low histidine contents, higher levels in casein kinase II and eukaryotic translation initiation factor 3 may contribute to binding of these proteins to cisplatin.

**Table 1:** Cisplatin-binding proteins identified in excised gel bands.

Protein name (gene name) [gel region]	Protein MW	Unique peptides	Unique spectra	Total spectra	Sequence coverage (%)	Histidine content (%)
Myosin-9 (Myh9)	226373	203	706	833	78.0	1.43
*Endoplasmin (Hsp90b1)	92477	74	346	361	69.2	1.25
**Transitional endoplasmic reticulum ATPase (Vcp)	89323	72	317	326	77.0	1.24
Heat shock protein HSP 90-alpha (Hsp90aa1)	84789	69	276	490	63.2	1.64
Heat shock protein HSP 90-beta (Hsp90ab1)	83282	42	254	531	66.6	1.80
Calreticulin (Calr)	47996	36	314	314	70.2	1.68
60S ribosomal protein L5 (Rpl5)	34402	36	223	223	68.7	1.68
Protein disulfide-isomerase A4 (Pdia4) [a]	71983	48	251	251	61.0	1.87
78 kDa glucose-regulated protein (Hspa5) [a]	72423	45	162	176	57.4	0.92
Elongation factor 1-gamma (Eef1g) [b]	50062	27	94	94	51.3	1.83
Elongation factor 1-alpha (Eef1a1) [b]	50115	24	63	63	49.6	2.16
Lupus La protein homolog (Ssb) [c]	47757	37	99	99	59.8	2.65
Obg-like ATPase 1 (Ola1) [c]	44731	33	128	128	73.0	2.27
COP9 signalosome complex subunit 4 (Cops4) [d]	46286	29	71	71	82.3	2.71
Casein kinase II subunit alpha (Csnk2a1) [d]	45135	22	62	66	58.1	4.09
Nucleophosmin (Npm1) [e]	32561	25	80	80	70.5	1.71
Eukaryotic translation initiation factor 3 subunit I (Eif3i) [e]	36462	21	81	81	80.6	3.38

Protein and gene names are those used in the SwissProt database. Gel regions of the identified proteins are indicated in Fig. 6B. “Protein MW” is the molecular weight of the protein sequence from the SwissProt database. “Total spectra” is the number of MS/MS spectra matched to the protein sequence. “Unique spectra” is the number of MS/MS spectra that are not shared between other protein sequences in the database. “Unique peptides” is the number of different amino acid sequences matched. “Sequence coverage” is the percentage of the total protein sequence matched to assigned MS/MS spectra. “Histidine content” is the percentage of the histidine residues in the total amino acids of each protein. Myosin-9 is the same as myosin IIA. *Endoplasmin is the same as GRP94. **Transitional endoplasmic reticulum ATPase is the same as VCP. The seven identified isoforms of tubulin are not included in the table.

### HSP90α expression is low in kidney proximal tubule cells

**Figure 7:**
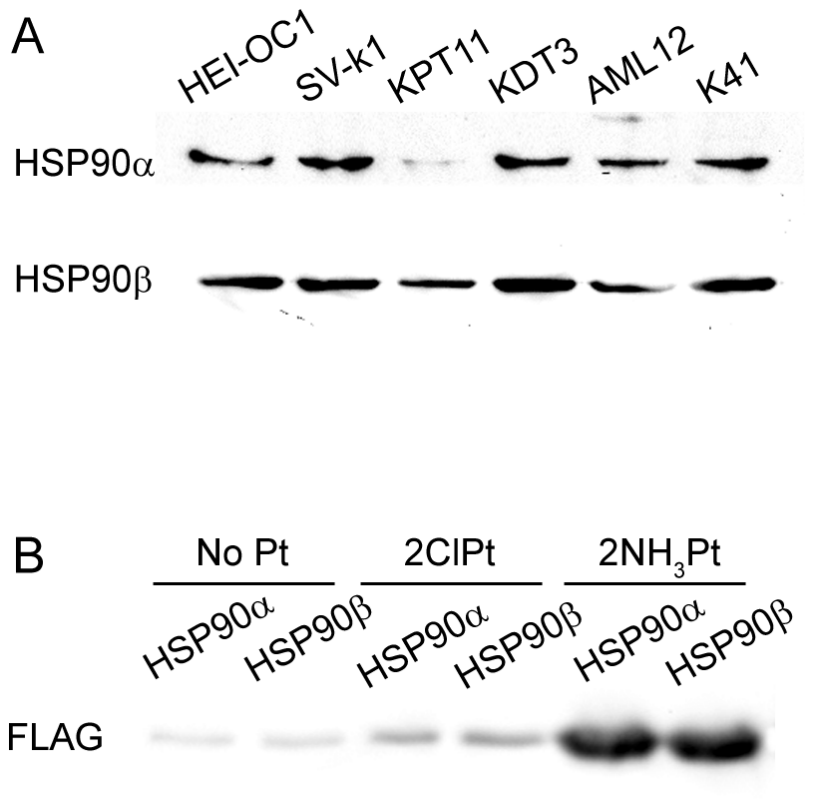
Protein expression and cisplatin-binding of HSP90α and HSP90β. (A) HSP90 protein expression analysis. Total cell lysates from cell lines used for pull-down assay were analyzed by Western blotting, using antibodies for HSP90α and HSP90β. KPT11 cell sample showed very low expression of HSP90α. (B) Pt-agarose pull-down assay using 293T cells transfected with FLAG-HSP90α or -HSP90β. 2NH_3_Pt-agarose pulled down the two HSP90 isoforms with similar amounts.

Since the 85 kDa band shown in Fig. 6B was weak in KPT11 cells, we wanted to determine which of HSP90α and HSP90β that was low in expression in these cells. Western blotting analysis on total cell lysate revealed that HSP90α protein expression was very low in KPT11 cells compared to the other cell lines, although HSP90β expression was also relatively low (Fig. 7A), To determine if the binding affinity for cisplatin was different between HSP90α and HSP90β, Pt-agarose pull-down assay was performed using cell lysate from 293T cells transfected with FLAG-HSP90α and -HSP90β, and analyzed by Western blotting. 2NH_3_Pt-agarose pulled down both FLAG-tagged HSP90 proteins at similar amounts, which suggests that there was no significant difference in binding affinity for cisplatin. 2ClPt-agarose also pulled down these proteins, but at levels nearly as low as agarose without Pt, suggesting that HSP90 binding to 2ClPt-agarose is likely non-specific.

### HSP90α expression contributes to cisplatin-induced cytotoxicity

**Figure 8:**
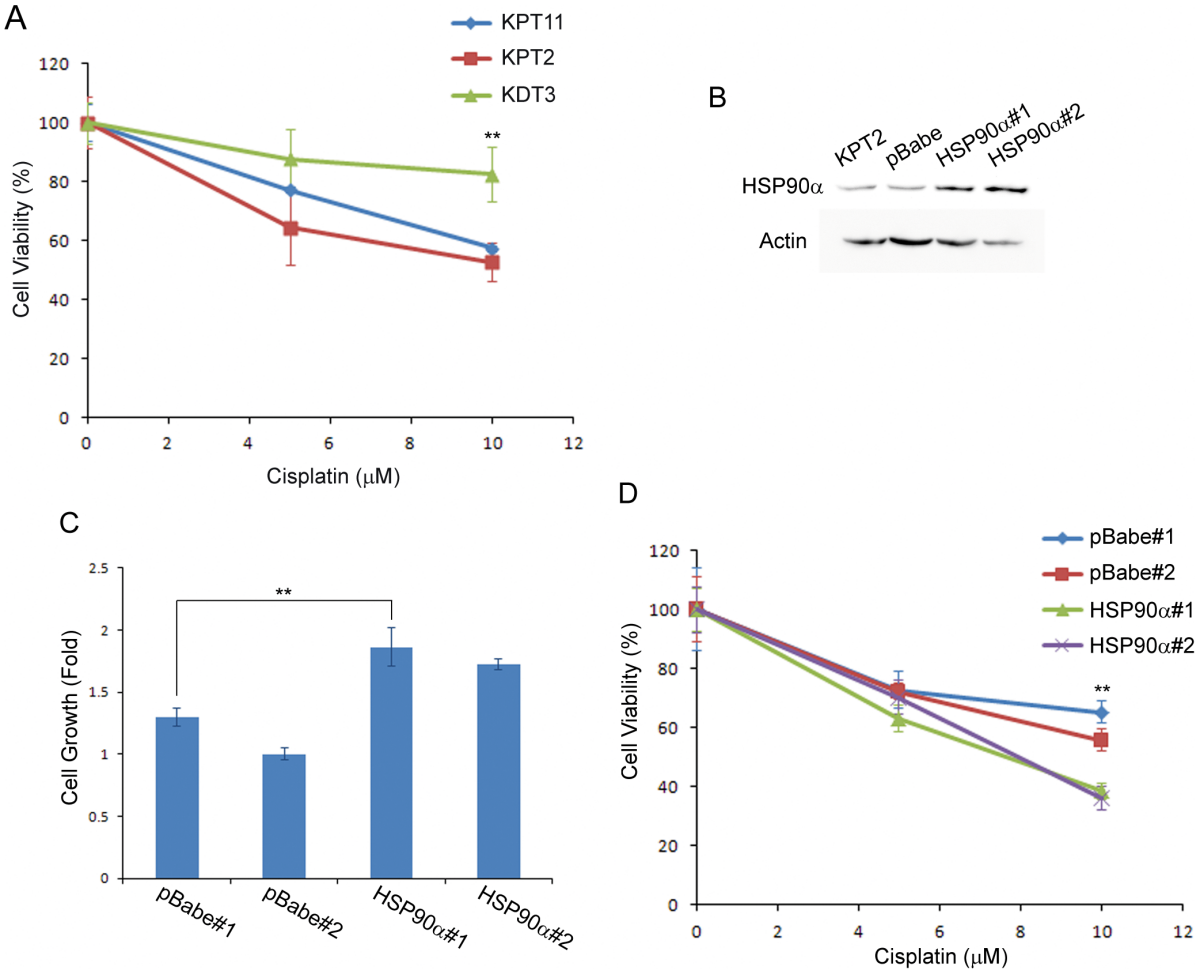
HSP90α expression and cell viability. (A) MTT assay on cells treated with cisplatin for 2 days showed that kidney proximal tubule cells (KPT11 and KPT2) had lower viability compared to kidney distal tubule KDT3 cells (**P<0.01 between either one of proximal tubule cell lines and KDT3). (B) Western blotting confirmed that KPT2-HSP90α cell lines (HSP90α#1 and #2) express higher levels of HSP90α compared to parental KPT2 or KPT2 with empty pBabe vector control. (C) Cellular growth for 2 days was assessed by MTT assay, and KPT2-HSP90α cell lines showed faster cellular growth compared to KPT2-pBabe cell lines (**P<0.01). (D) Cisplatin treatment for 2 days showed that KPT2-HSP90α cell lines had lower viability compared to KPT2-pBabe cell lines (**P<0.01 between either one of KPT2-HSP90α and either one of KPT2-pBabe).

As a first step to determine if HSP90α has a role in cisplatin-induced cytotoxicity, we compared mouse kidney cell lines for sensitivity to cisplatin treatment. As expected from kidney proximal tubule as a major cisplatin toxicity site, proximal tubule KPT11 and KPT2 cells showed lower cell viability compared to distal tubule KDT3 after 2 days of cisplatin treatment (Fig. 8A). To determine how HSP90α expression affects cisplatin-induced cytotoxicity, we attempted to use RNA interference and knocked down HSP90α expression in KDT3; this resulted in the death of most cells transfected with HSP90α siRNA, even without cisplatin treatment (unpublished data). Therefore, we used an alternative approach, and generated stable cell lines derived from KPT2 cells with higher expression levels of HSP90α (KPT2-HSP90α) by retroviral gene transfer. Western blotting confirmed that these KPT2-HSP90α cells express higher levels of HSP90α, compared to parental KPT2 and KPT2 with empty pBabe vector control (KPT2-pBabe) cell lines (Fig. 8B). The KPT2-HSP90α cells (HSP90α#1 and #2) exhibited faster cellular growth compared to KPT2-pBabe cell lines (pBabe#1 and #2), as assessed by MTT assay (Fig. 8C). Strikingly, cisplatin treatment on these cell lines for 2 days revealed that KPT2-HSP90α cells were more sensitive to cisplatin compared to KPT2-pBabe cells, suggesting that HSP90α expression contributes to cisplatin cytotoxicity.

## Discussion

Although cisplatin-DNA adduct formation and subsequent apoptosis mechanisms in cancer have been well documented, its toxicity mechanisms in the cochlea and kidney are not well understood. Oxidative stress induced by aquated cisplatin likely causes ototoxicity and nephrotoxicity. However, aquation of carboplatin is much slower than cisplatin, yet this drug is still toxic to the cochlea and kidney. We hypothesized that cochlear- and kidney-specific proteins bind to cisplatin and carboplatin at the two amino groups, and that this drug-protein interaction induces cytotoxicity. To identify such proteins, we decided to use the drug-agarose conjugation technique that we used previously to identify gentamicin-binding proteins [26], [28]. This method took advantage of existing cell lines generated from mouse cochlea or kidney tissue, to avoid problems with very limited amounts of tissues available from each animal, and the extended time needed to extract proteins from *in vivo* tissues that can be a major cause of protein degradation. Since the amino groups of cisplatin are -NH_3_ covalently coordinated to the platinum atom, these amino groups cannot be used for conjugation to agarose. In addition, conjugation of agarose to a covalently coordinated amino group of cisplatin would make proteins inaccessible to cisplatin at this site. Therefore, we synthesized platinum complexes that contain another free amino group for conjugation to agarose.

Pull-down assays using Pt-agarose conjugates revealed that the amino group site of cisplatin has much higher affinities for protein binding compared to the chloride site. Although aquated cisplatin binds to antioxidant proteins at the chloride site, it is likely these proteins are not abundantly expressed in the cell lines tested, because we only detected several weak protein bands by SDS-gel analysis. Unlike the chloride site of cisplatin which is the binding site for DNA and antioxidants [7], [14], the role of the amino group site has been unknown. It is, therefore, significant that we identified several binding proteins to the amino group site which may influence the efficacy of cisplatin-induced toxicity.

In the 2NH_3_Pt-agarose pull down samples, we expected to see some differences among cell lines in Coomassie blue-stained protein bands. Specifically, we expected more proteins in HEI-OC1 and KPT11 samples compared to the others because cochlear hair cells and kidney proximal tubule cells are major sites of cisplatin-induced toxicity in these organs. However, we did not find significant differences among the mouse cell lines we tested. It is possible that these cell lines have gained some common characteristics that the original tissues do not. Gene expression associated with rapid cellular proliferation likely is a major factor. We will discuss several of the most abundant proteins that bound to 2NH_3_Pt-agarose and their relevance to cisplatin-induced cytotoxicity and/or tumor resistance to cisplatin treatment.

### Myosin IIA

Myosin IIA, also known as myosin-9, is an isoform of class II non-muscle myosins. Myosin IIA regulates cell motility and maintains a balance between actomyosin and microtubule systems [37]. In the cochlea, this protein is expressed in sensory hair cells and its mutation is responsible for syndromic and nonsyndromic hearing loss [38], [39]. Although exact functions of myosin IIA in hair cells are unclear, localization of this protein within the mitochondria suggests that it has an important role in maintaining mitochondrial function of hair cells [40].

### GRP94

Glucose-regulated protein 94 (GRP94), also known as endoplasmin or HSP90B1, is a chaperone protein in the endoplasmic reticulum (ER). This is the most abundant protein in the ER, accounting for 5–10% of all the lumenal proteins [41]. However, its function is not as well-defined as most other ER-localized proteins. Unlike many other molecular chaperones in the ER, GRP94 interacts with a limited set of protein substrates. GRP94 binds to only several different proteins in the secretory pathway, including Ig chains, MHC class II, thyroglobulin, erbB2, a herpes virus glycoprotein, and apolipoprotein B [42]. GRP94 can also bind to peptides, giving rise to GRP94's potential as a cancer vaccine to induce a T cell response [42]. Physiological functions of GRP94 in the ER are thought to be similar to its cytosolic counterpart HSP90.

### HSP90

Heat shock protein 90 (HSP90) is a cytoplasmic molecular chaperone. The α form is inducible and β form is constitutively expressed, and these isoforms are highly similar in sequence and function. HSP90 is generally thought to function in assisting protein folding and maintenance, and degradation of misfolded proteins. Recent advances in cancer biology have revealed critical roles of HSP90 in cancer cells, where HSP90 protects mutated and overexpressed proteins encoded by oncogenes from degradation, facilitating cancer cell survival [43].

HSP90 has previously been identified as a cisplatin-binding protein [44]. In this study, we expected that HSP90 was protective against cisplatin treatment, and that kidney proximal tubule cells were sensitive to cisplatin toxicity because of low expression of HSP90. However, cell viability experiments using kidney proximal tubule cell lines with exogenous HSP90α expression suggested that HSP90α expression contributes to cisplatin-induced cytotoxicity. Based on the accelerated cellular growth in these cell lines, this raises a possibility that HSP90α-induced faster cellular growth is the major cause of enhanced sensitivity to cisplatin. Perhaps in some cancer cells, overexpression of HSP90α makes these cells more susceptible to cisplatin treatment. This possibility needs to be carefully examined further and differences between cancer and normal cells should be taken into consideration. Although the significance of cisplatin binding to HSP90 is still unclear, low expression of HSP90α in kidney proximal tubule cells suggests that HSP90 could be protective against cisplatin *in vivo*, where cells do not rapidly proliferate. We also predict that HSP90 is protective against cisplatin-induced ototoxicity, since heat shock proteins including HSP32 have been shown to inhibit ototoxicity [45]. In support of this possibility, cisplatin binding to HSP90 reduces its chaperone activity, which would further decrease cellular viability, causing nephrotoxicity [46].

### Calreticulin

Calreticulin is an ER-localized protein that specifically binds to glycoproteins. This is also a molecular chaperone assisting in protein folding and trafficking misfolded proteins for degradation [47]. Identification of calreticulin as a cisplatin-binding protein has potential significance, because calreticulin is also a gentamicin-binding protein, and reduces gentamicin-induced cytotoxicity [28], [48]. Since both cisplatin and gentamicin induce toxicity in the cochlea and kidney and calreticulin is expressed in these tissues [28], the function of the protein may be highly relevant to cisplatin-induced toxicity in these tissues.

### GRP78

We identified glucose-regulated protein 78 (GRP78), also known as BiP or HSPA5, with relatively low abundance. This protein also functions as a molecular chaperone in the ER, like GRP94 and calreticulin, and GRP78 expression levels have been correlated with cisplatin resistance in certain cancers [49], [50]. Overexpression and/or cisplatin-induced up-regulation of GRP78 significantly reduce the antineoplastic efficacy of cisplatin. Unlike GRP94, this protein binds to a wide range of proteins and is referred to as the master regulator of the ER [47].

### VCP

Valosin-containing protein (VCP), also known as p97 or transitional endoplasmic reticulum ATPase, is an ATPase of the AAA family (ATPases associated with diverse cellular activities), and is a key player in various ubiquitin-dependent processes [51]. VCP expression has also been correlated with cancers, including non-small cell lung carcinoma and hepatocellular carcinoma [52], [53]. Significantly, VCP has been shown to promote tumor suppressor protein 53BP1 recruitment to DNA damage sites to repair the damage, providing defense against cancer for normal cells [54].

### Ribosomal protein L5

Ribosomes consist of a small 40S subunit and a large 60S subunit. We identified L5, a part of the 60S subunit, to be a cisplatin-binding protein. Although the role of this protein in cancer is highly speculative, overexpression of ribosomal protein L5 has been identified in several colorectal cancer cases [55], which suggests a potential significance of the protein in tumor development or resistance to antineoplastic drugs.

### Conclusions

We synthesized agarose conjugates of two different platinum complexes and performed pull-down assays to identify cisplatin-binding proteins. While most of the proteins we identified were not specific to the cochlea or kidney, we found that these cisplatin-binding proteins to be highly relevant to cisplatin-induced cytotoxicity. Some of these proteins likely contribute to cisplatin sensitivity (e.g. HSP90) or resistance (e.g. GRP78) of cancers by binding to cisplatin. The proteins identified here by this technique could represent a minority of highly expressed proteins and not the large number of proteins which are likely more poorly expressed. Nevertheless, this study provides a method to identify novel cisplatin-binding proteins and determine how their dysregulation can contribute to cisplatin-induced toxicity as well as sensitivity or resistance of tumor cells to cisplatin.
